# Primary hepatic mucosa-associated lymphoid tissue lymphoma: A case report and literature review

**DOI:** 10.1097/MD.0000000000015034

**Published:** 2019-03-15

**Authors:** Huazhi Xie, Jian Lv, Yong Ji, Xinjian Du, Xin Yang

**Affiliations:** Department of Hepatobiliary Surgery, Jingjiang People's Hospital, Affiliated to Medical College of Yangzhou University, Taizhou, China.

**Keywords:** case report, hepatic tumor, liver, lymphoma, mucosa-associated lymphoid tissue

## Abstract

**Rationale:**

Primary hepatic mucosa-associated lymphoid tissue (MALT) lymphoma is a rare disease, and there is no consensus yet on the treatment modalities. Here, we report a new case of MALT lymphoma and review the current literature on this disease.

**Patient concerns:**

A 73-year-old man was admitted to our department following the incidental finding of a solitary 1.8-cm diameter mass in the liver.

**Diagnosis:**

Microscopic findings identified the mass as a tumor with infiltration of diffuse atypical B lymphocytes. Immunohistochemical analysis showed positivity for CD20 and CD79a, and negativity for CD3 and CD5. These collective data led to the diagnosis of primary hepatic MALT lymphoma.

**Interventions:**

The tumor was removed by surgical resection. The patient refused additional treatment after the surgery.

**Outcomes:**

At the time of writing this report, the patient has been disease free for 6 months postsurgery.

**Lessons:**

Review of the previously published case reports on this rare tumor type indicates that in addition to chronic liver inflammation due to infection or other reasons, genetic aberrations can also contribute to the development of hepatic MALT lymphoma. Additionally, IgH rearrangement is a good genetic hallmark of this tumor. Owing to no specific clinical or radiologic features to define the disease profile for diagnosis, surgery may be a good choice for both diagnosis and therapy if the patient's condition permits.

## 1 Introduction

Primary hepatic mucosa-associated lymphoid tissue (MALT) lymphoma is a rare disease. It was 1st described by Isaacson et al^[1]^ in 1995 as an extranodal marginal zone B-cell lymphoma with low-grade malignancy. However, subsequent reports have indicated the involvement of organs such as the stomach, lungs, parotid glands, thyroid, and on very rare occasions the liver.^[2,3]^ To date, the PubMed database includes only 36 articles (all case reports) describing primary MALT lymphoma of the liver. Unfortunately, no definitive etiologic profile has been established for this disease, and there is no consensus on the best approach for its management. In this report, we discuss our clinical experience of treating a patient with primary hepatic MALT lymphoma. We also present our findings from a systematic review of the related PubMed literature, discussing the possible treatment modalities for this rare disease.

## 2 Case report

A 73-year-old man was admitted to our department following the incidental finding of a focal liver mass in computed tomography (CT) scan. The patient reported no symptoms, other than upper abdominal discomfort. He had no significant medical history and no remarkable family history. Physical examination did not show any abnormalities. Findings for all clinical laboratory tests conducted at our institution, including those for the carcinoembryonic antigen and α-fetoprotein tumor markers, were within the normal range (Table 1). However, he tested positive for serum markers of hepatitis B virus (HBV) infection (i.e., antigens and antibodies to hepatitis B surface protein, hepatitis B e protein, and hepatitis B core protein), with a viral load of 1.99 × 10^3^ IU/mL, but was negative for hepatitis C virus and human immunodeficiency virus.

**Table 1:**
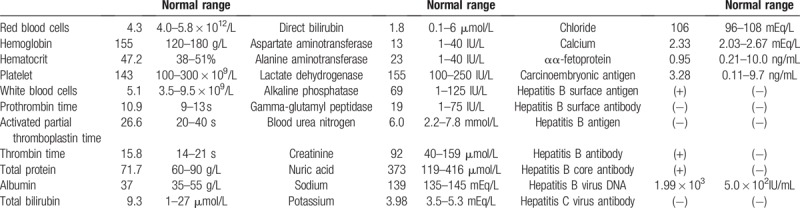
Laboratory findings upon admission.

**Figure 1:**
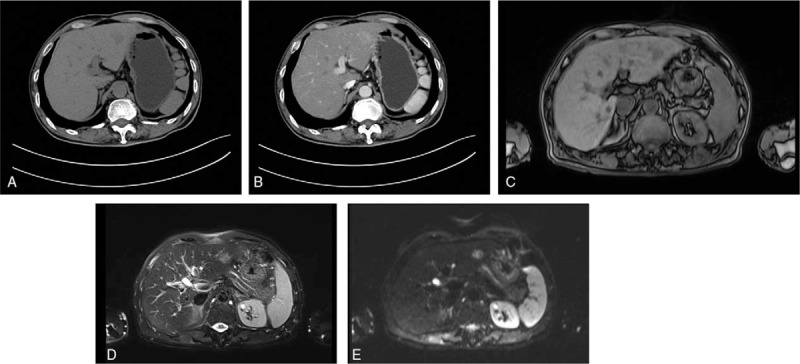
Computed tomography (CT) and magnetic resonance imaging (MRI) findings. Shown here is the mass with (A) low-signal intensity on plain CT and (B) slightly high-signal intensity on contrast-enhanced CT in the arterial phase. In contrast-enhanced MRI, the mass shows (C) low-signal intensity on T1-weighted imaging, and high-signal intensity on (D) T2-weighted imaging and (E) diffusion-weighted imaging.

The mass in the left hepatic lobe (segment II) was detected on plain CT as a hypodense lesion (Fig. 1A). Contrast-enhanced CT and gadopentetate dimeglumine-enhanced magnetic resonance imaging (MRI) were performed for precise imagistic evaluation. The CT revealed the mass as a faint enhancement during the arterial phase (Fig. 1B). However, in the MRI, the lesion appeared to have low intensity on T1-weighted imaging (Fig. 1C), slightly high intensity on T2-weighted imaging (Fig. 1D), and intense restriction of diffusion on diffusion-weighted imaging (Fig. 1E).

**Figure 2:**
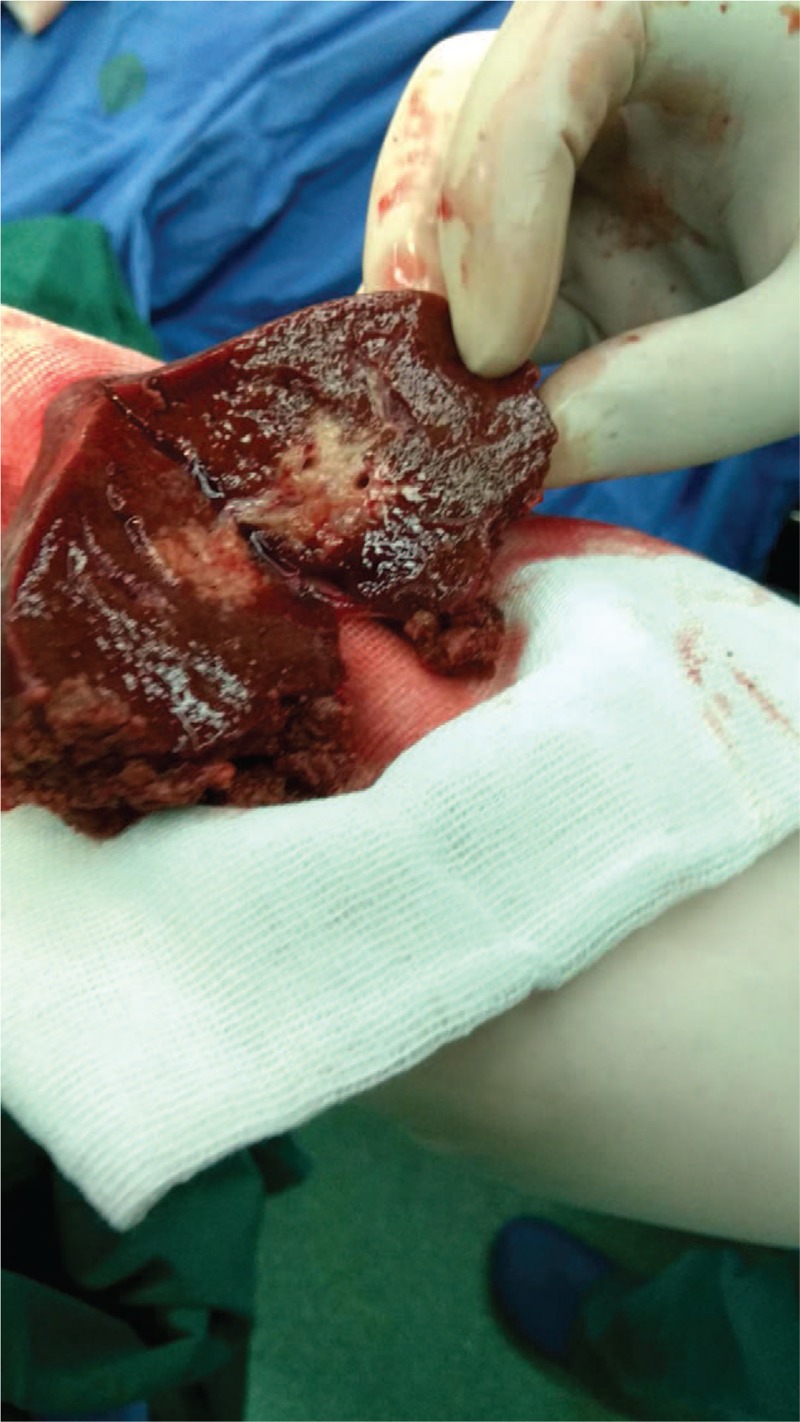
Gross appearance of the resected mass. The nodular tumor is whitish in color and 1.8 cm in size.

**Figure 3:**
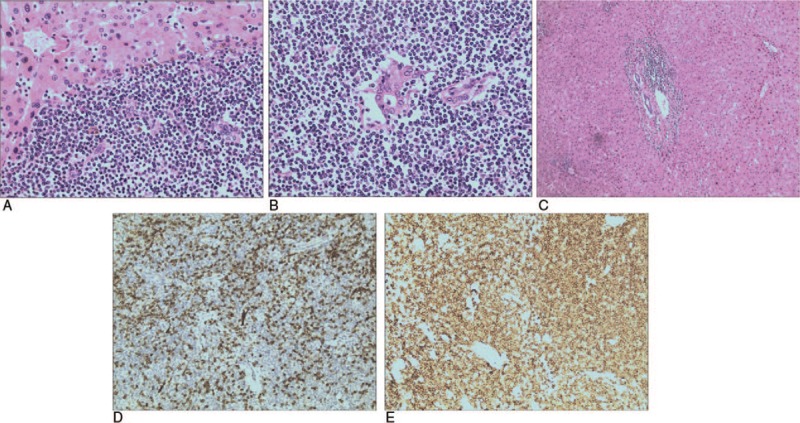
Histologic characteristics of the resected mass. Hematoxylin-eosin staining shows (A: ×20) diffuse infiltration of the hepatic lobule and (C: ×10) the hepatic portal area by atypical lymphocytes. (B: ×20).Small to medium-sized lymphoid cells can be seen infiltrating into the bile duct, forming lymphoepithelial lesions. Immunohistochemistry shows the lymphocytes are (D: ×20) diffusely negative for CD3 and (E: ×20) positive for CD20 antibodies.

Based on the clinical and radiologic findings, the lesion was diagnosed as malignant. The patient consented to undergo a laparoscopic left lateral segment liver resection (segments II–III). The gross finding following the resection was a white-colored, 1.8 cm, nodular tumor mass (Fig. 2). Histologic analysis of the resected tissue revealed a large number of atypical lymphocytes diffusely infiltrating the hepatic lobule and the portal area and lymphoepithelial lesions with small to medium-sized lymphocytes on some of the bile capillaries. Immunohistochemical analysis showed that the lymphocytes were positive for CD20, Ki67, PAX-5, BCL-2, CD79a, CD21, and CD23, but negative for Bcl-6, CD3, CD5, CD10, CD43, CD56, CD138, MUM1, and cyclin D1 (Fig. 3).

Based on the above-mentioned pathologic features, the patient was diagnosed with primary hepatic MALT lymphoma, specifically a low-grade malignant extranodal marginal zone B-cell lymphoma. The patient refused any additional treatment after the surgery and remained disease free throughout the 6 months of follow-up (at the time of writing of this report).

## 3 Discussion

The MALT lymphoma was 1st described by Isaacson and Wright in 1983.^[4]^ Today it is described as an extranodal marginal zone B-cell lymphoma, a type of non-Hodgkin lymphoma. The incidence of MALT lymphoma is rare, representing only 7% to 8% of all non-Hodgkin lymphoma cases.^[5]^ It involves a wide variety of organs, such as the gastrointestinal tract, thyroid, lung, parotid glands, breast, and liver.^[2,3]^ While the most common primary sites are the stomach, thyroid, and lungs, the incidence of MALT lymphoma in the liver is extremely rare.^[2,3]^

Little is known about the clinical features and optimal treatment modalities for hepatic MALT lymphoma. A systematic review of the PubMed database using the keywords “((MALT[Title]) OR mucosa-associated lymphoid tissue[Title]) AND ((liver[Title]) OR hepatic[Title])” yielded only 45 potentially relevant articles. After reviewing the titles and abstracts for relevance to primary hepatic MALT lymphoma, we had 36 articles. Exclusion of articles without full-text (n = 1, English language) and English language translation (n = 1, Japanese; n = 2, French) led to a final count of 31 articles describing 46 cases^[1,6–35]^ besides the case we are presenting (Tables 2 and 3).

**Table 2:**
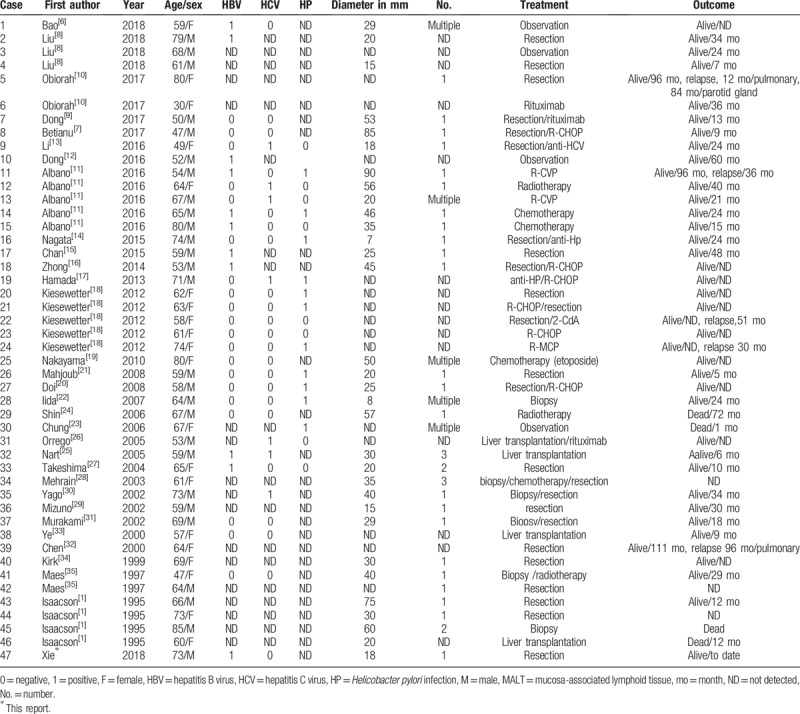
Reported cases of hepatic MALT lymphoma.

**Table 3:**
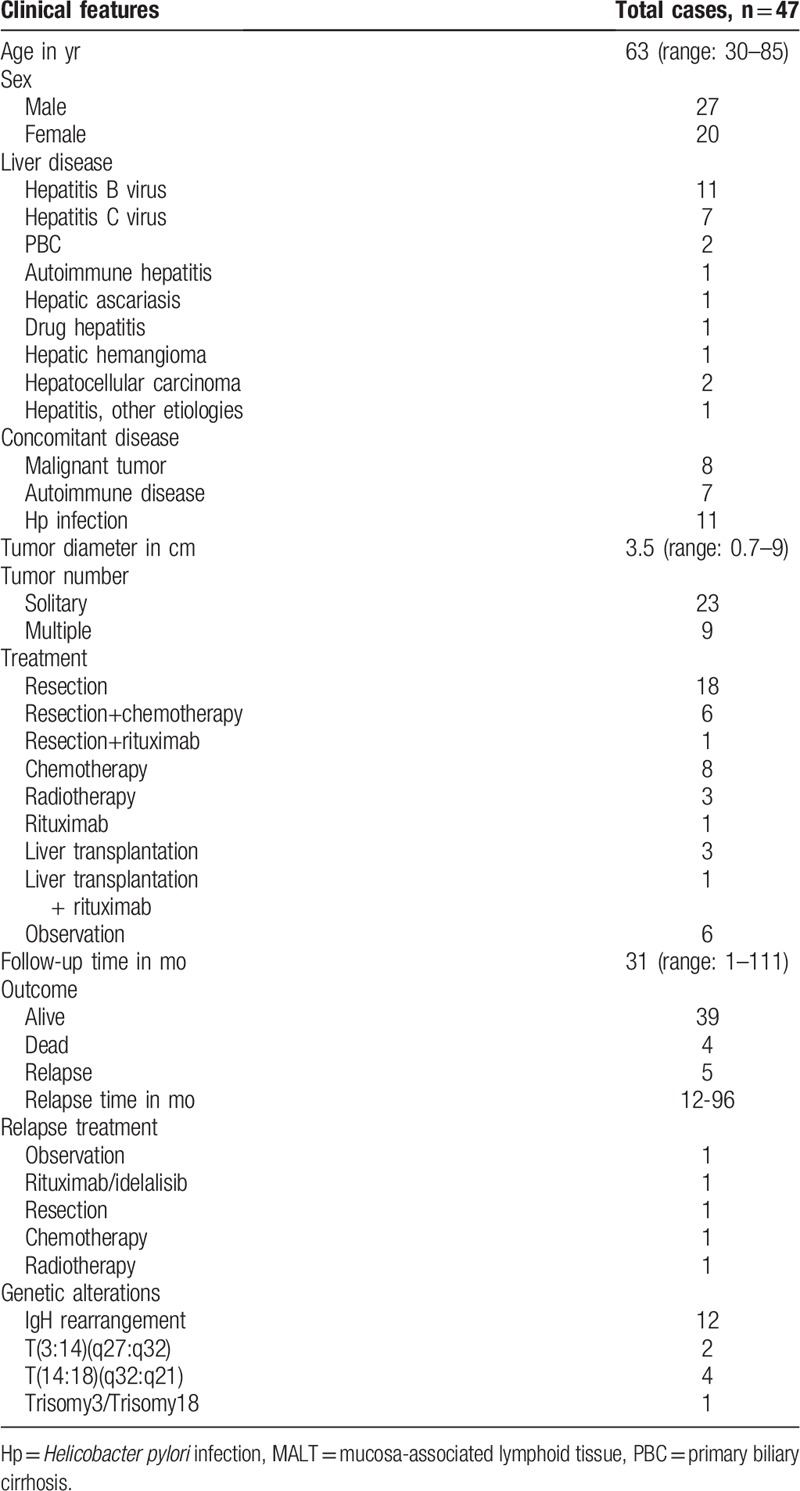
Clinical features of the reported cases of hepatic MALT lymphoma, including the present case.

Nonhepatic MALT lymphoma usually arises in the presence of chronic inflammation, due to either an infectious or autoimmune process. However, the etiology of hepatic MALT lymphoma is still unclear. The 47 cases considered in this paper (Table 3) included 26 patients (55%) with various concomitant basic liver diseases, including HBV (n = 11), hepatitis C virus (n = 7), primary biliary cirrhosis (n = 2), hepatic cell carcinoma (n = 2), and other liver diseases (n = 4). Our patient also had HBV infection. Collectively, these data support that chronic liver inflammation due to infection or other reasons might contribute to the development of hepatic MALT lymphoma.

Different nonhepatic MALT lymphomas have also been associated with inflammatory conditions, such as gastric MALT lymphoma with *Helicobacter pylori* infection,^[36]^ thyroid MALT lymphoma with Hashimoto disease,^[37]^ and thymic MALT lymphoma with Sjögren syndrome.^[38]^ Thus, a plausible hypothesis is that chronic inflammation can stimulate the development of MALT lymphoma, with a nodule-like structure of the ileum being formed and marginal zone cells gradually proliferating under the inflammatory stimuli to ultimately develop into a MALT lymphoma. However, it is important to remember that 45% of the cases from the literature had no concomitant liver diseases, and they cannot be explained by the above theory.

A tumor is essentially a genetic disease, and 4 chromosomal ectopic recombinations have been identified in MALT lymphomas.^[39]^ According to our literature review (Table 3), genetic aberrations such as IgH rearrangement (12 cases),^[10,15,18,19,21,34,35]^ including 2 cases of T(3:14)(q27:q32), 4 cases of T(14:18)(q32:q21), and 1 case of Trisomy3/Trisomy18 have been reported in MALT lymphomas. Among these 12 cases, while 4 patients had *H pylori* infection, 1 had HBV infection, and the remaining 7 patients had no known infections. Thus, besides infections, genetic aberrations are also likely to be involved in the development of hepatic MALT lymphoma, and IgH rearrangement might be a good genetic hallmark of this disease.

The 47 total cases of hepatic MALT lymphoma included in this review (Table 3), included 27 men and 20 women, with a mean age of 63 years, which is consistent with the findings reported in earlier reviews by Nagata et al^[14]^ (64 years) and Dong et al^[9]^ (62 years). The clinical presentations in these cases varied, ranging from nonsymptomatic to end-stage liver disease. However, most of the patients experienced no symptoms and were incidentally identified during clinical examinations or surgical explorations.

The majority (72%) of the patients with no symptoms (n = 32) presented with a solitary mass. Of these, 17 patients underwent surgical resection, and 3 each received chemotherapy and radiotherapy. The remaining 9 patients presented with multiple masses, and while 2 of them underwent surgical resection with or without chemotherapy, 1 underwent liver transplantation, 2 received chemotherapy, and the remaining 4 patients did not receive any treatment.

The tumors ranged in size from 0.7 to 9.0 cm in diameter, with an average diameter of 3.5 cm. No distinctive radiologic characteristics were apparent. The hepatic MALT lymphomas appeared to be hypoechoic masses in 8 cases (ultrasound studies),^[7–9,11,20,27,28,31]^ hypodense lesions in another 8 cases (CT findings),^[6,7,11,17,30,31]^ and slightly enhanced in 5 cases (with contrast administration).^[6,17,20,28,29]^ The MRI revealed the masses appearing as low density T1-weighted images in 8 cases,^[9,11,17,27,28]^ as high-density T2-weighted images in 9 cases,^[7,9,11,13,17,27,28]^ and with restricted diffusion on diffusion-weighted imaging in 1 case.^[7]^ Our patient also showed these radiologic features on CT and MRI. However, these imaging features are similar to those for other malignant tumors of the liver, such as hepatocellular carcinoma, intrahepatic cholangiocellular carcinoma, and metastatic tumor, making it difficult to differentiate them from hepatic MALT lymphomas.

In our present case, the patient was a 73-year-old man with HBV infection, and the tumor was found incidentally during a CT scan for upper abdominal discomfort. The solitary mass detected was 1.8 cm in diameter. The patient underwent surgical resection due to a suspicion of hepatocellular carcinoma, which led to the fortuitous diagnosis of primary hepatic MALT lymphoma. As of when writing this report, the patient has remained disease free without any additional therapy (albeit only 6 months have elapsed) since the surgical treatment.

To date, no optimal therapeutic strategies have been established for the clinical management of primary hepatic MALT lymphomas, because of the small number of patients worldwide. With regards to the indolent and favorable prognosis of this tumor (Table 3), in most of the reported cases (25/47, 53%) the patients underwent surgical resection with or without chemotherapy or rituximab. Of these, 17 patients (17/47, 36%) had a solitary tumor mass. While 4 patients (4/47, 8.5%) underwent liver transplantation with or without rituximab, 8 of them (8/47, 17.0%) received only chemotherapy, 3 (3/47, 6.4%) received only radiotherapy, 1 (1/47, 2.1%) received only rituximab, and 6 patients (6/47, 12.8%) did not receive any treatment. Most cases had positive outcomes, with a mean follow-up time of 31 months (range: 1–111 months). According to the reports, only 4 out of the 47 patients (8.5%) died before case reporting, which included one patient each who refused any treatment and had sepsis, had postoperative complications following a liver transplant, had an aortic aneurysm rupture, and had an undefined lung disease.

Recurrence was reported in 5 cases (5/47, 11%) with relapse times ranging from 12 to 96 months. These cases included 1 each involving the pulmonary system, pulmonary system 1st and the parotid gland in a 2nd recurrence, and liver. The involved organs for the remaining 2 cases were not reported. Of these 5 cases, 1 patient refused any further treatment. The patient who experienced 2 relapses received rituximab after the 1st relapse, which resulted in complete remission for 84 months, and then received idelalisib after the second relapse, again achieving complete remission. The remaining 3 patients underwent either resection, chemotherapy or radiotherapy only, and all of them achieved complete remission.

Considering the data for the 47 collective cases reported, hepatic MALT lymphoma is an extremely rare disease, tending to be a solitary and small tumor, mostly occurring in elderly people. The etiology of hepatic MALT lymphoma is still unclear, though chronic liver inflammation due to infections or other reasons, and genetic aberrations might be contributory factors to its development. Owing to the general findings of no specific clinical presentation or distinctive radiologic features, it is often difficult to make a definite diagnosis of hepatic MALT lymphoma before tissue resection and histologic confirmation.

To date, no standard management modalities have been recommended for this rare disease. Based on the National Comprehensive Cancer Network (commonly known as the “NCCN”) guidelines for non-Hodgkin lymphomas,^[39]^ our review of relevant literature, and on the disease's indolent characteristics, the reasonable choices for its management include surgery, radiotherapy, chemotherapy, targeted therapy alone or in combination, or even simply observation. Since it is challenging to diagnose hepatic MALT lymphoma before the operation and there are concerns regarding needle metastasis of a malignant tumor, surgery is probably the best choice for both diagnosis and therapy, as long as the patient's physical condition permits. However, the treatment choice should be based on the size, location, and number of the tumors as well as the patient's performance status. Additional reports will enhance our knowledge of this rare disease and help improve its management.

## Acknowledgments

The authors thank Dr Hua Liu, Jue Wang, and Jing Chen for their technical support and for providing the pathologic pictures.

## Author contributions

**Conceptualization:** Jian Lv.

**Data curation:** Yong Ji.

**Formal analysis:** Yong Ji.

**Supervision:** Xin Jian Du, Xin Yang.

**Writing - Original Draft:** Hua Zhi Xie.

**Writing - Review & Editing:** Hua Zhi Xie, Jian Lv.
